# Role of ^99m^Tc-Sulesomab Immunoscintigraphy in the Management of Infection following Deep Brain Stimulation Surgery

**DOI:** 10.1155/2011/817951

**Published:** 2011-10-17

**Authors:** Raquel Real, Paulo Linhares, Hélder Fernandes, Maria José Rosas, Miguel F. Gago, Jorge Pereira, Rui Vaz

**Affiliations:** ^1^Department of Neurology, Hospital de São João, Alameda Prof. Hernâni Monteiro, 4200 Porto, Portugal; ^2^Faculty of Medicine, Universidade do Porto, Alameda Prof. Hernâni Monteiro, 4200 Porto, Portugal; ^3^Department of Neurosurgery, Hospital de São João, Alameda Prof. Hernâni Monteiro, 4200 Porto, Portugal; ^4^Department of Nuclear Medicine, Hospital de São João, Alameda Prof. Hernâni Monteiro, 4200 Porto, Portugal

## Abstract

Infection constitutes a serious adverse event in patients submitted to deep brain stimulation, often leading to removal of the device. We set to evaluate the potential role of immunoscintigraphy with ^99m^Tc-labelled antigranulocyte antibody fragments (^99m^Tc-sulesomab) in the management of infection following DBS. ^99m^Tc-sulesomab immunoscintigraphy seems to correlate well with the presence and extent of infection, thus contributing to differentiate between patients who should remove the hardware entirely at presentation and those who could undergo a more conservative approach. Also, ^99m^Tc-sulesomab immunoscintigraphy has a role in determining the most appropriate timing for reimplantation. Finally, we propose an algorithm for the management of infection following DBS surgery, based on the results of the ^99m^Tc-sulesomab immunoscintigraphy.

## 1. Introduction

Deep brain stimulation (DBS) is an effective treatment option for a number of neurological disorders, including movement disorders, pain, and epilepsy [1–4]. Following DBS procedures, skin erosion and/or infection can occur, most commonly at the connector site [5, 6]. Infection constitutes a serious adverse event because it often leads to removal of the DBS system, with consequent loss of the clinical benefits of stimulation. Also, from an economical perspective, infection greatly adds to the costs of DBS treatment, as this complication often requires hospitalization, prolonged antibiotic therapy, and additional surgical procedures. Currently there are no guidelines that specifically address the issue of hardware-related infection. In a pooled analysis of ten studies concerning hardware-related complications of DBS, which included 922 patients, hardware removal was necessary in 80% of the patients who developed infections [5]. Nevertheless, reports of successful conservative treatment with antibiotics alone seem to indicate that removal of the stimulation device is not always necessary and that conservative treatment could be considered as first choice in cases of circumscribed extracranial hardware infections [7]. The evaluation of the true extent of the extracranial infection can be troublesome in clinical practice and the decision of which patients are candidates for a conservative medical approach in contrast to immediate surgical treatment is usually not sufficiently clear-cut. 

To address the issue of infection management following DBS surgery, we set to evaluate the potential role of immunoscintigraphy with ^99m^Tc-labelled antigranulocyte antibody fragments (sulesomab) in the development of a rational therapeutic approach to infection in patients with Parkinson's disease (PD) submitted to DBS. ^99m^Tc-sulesomab consists of monoclonal murine IgG antibody Fab' fragments labeled with technetium-99m that have affinity for a 42 kDa glycoprotein found on the surface of activated granulocytes (NCA-90 surface antigen). Immunoscintigraphy with ^99m^Tc-sulesomab has been used in different clinical settings to detect infectious foci, including bone and joint infection, soft tissue infection, and in the investigation of fever of unknown origin [8, 9]. There is currently no data reporting its efficacy in the context of DBS surgery.

## 2. Methods

This preliminary study involved eight consecutive PD patients with persistent device-related skin erosion and/or infection. All patients had been previously submitted to DBS of the subthalamic nuclei (STN) in a single center, in the period between November 2002 and October 2008. Bilateral electrode implantation in the STN was followed by impulse generator (Kinetra, Medtronic Inc.) placement in an infraclavicular subcutaneous pouch, in a single-stage procedure performed by the same team of surgeons. In addition, two of the patients underwent routine IPG replacement surgery in October 2008 and April 2009, respectively. Perioperative intravenous antibiotic prophylaxis was administered to all patients (vancomycin and ceftriaxone). After the surgery, routine follow-up visits were scheduled at regular intervals with a team of movement disorders specialists. 

The detection of skin erosion and/or infection prompted adequate treatment with antibiotics. In addition, some of the patients underwent partial removal and/or replacement of hardware components in an attempt to control infection. Because of persistent or recurrent wound dehiscence, immunoscintigraphy with ^99m^Tc-sulesomab was subsequently performed between July 2009 and April 2010. Planar scans and SPECT-CT were acquired 4 h and 24 h after injection of ^99m^Tc-sulesomab (Immunomedics Inc) using a dual-head gamma camera coupled with a low-power X-ray tube. Depending on the scan results, patients were further subjected to wound debridement alone or in combination with either partial or complete hardware removal. Microbiological examinations were performed from the skin exudates, when available. At followup, three patients repeated the scan in order to determine the feasibility of reimplantation of the stimulation hardware. Medical records of all patients were reviewed and data collected retrospectively. 

## 3. Results

Patients' characteristics, including the location of skin erosion and/or infection and the results of any surgical reinterventions preceding the immunoscintigraphy, are described in Table 1. The results of the ^99m^Tc-sulesomab immunoscintigraphy and the subsequent therapeutic procedures are summarized in Table 2, as well as patient outcome and followup.

### 3.1. Location of Skin Erosion and/or Infection

Of the six patients who developed hardware-related erosion and/or infection after the initial DBS surgery, five patients presented with wound dehiscence of the retroauricular incision, which in most cases occurred in isolation (patients 2–4). In the remainder of cases, the retroauricular wound dehiscence occurred in association with dehiscence at distinct locations, namely, the left frontal incision (patient 1) and the skin along the extension cables pathway in the neck (patient 5). One patient presented with isolated left frontal wound dehiscence (patient 6). Two additional patients presented with subclavicular wound dehiscence that developed after routine IPG replacement due to end of battery life. Patient 7 initially developed a hematoma at the IPG implantation site that was surgically addressed before persistent infection developed. In addition, patient 8 subsequently presented with wound dehiscence of the left frontal incision. *Staphylococcus aureus* was isolated from the wound exudate in most cases.

### 3.2. Pattern and Location of Tracer Uptake

**Figure 1:**
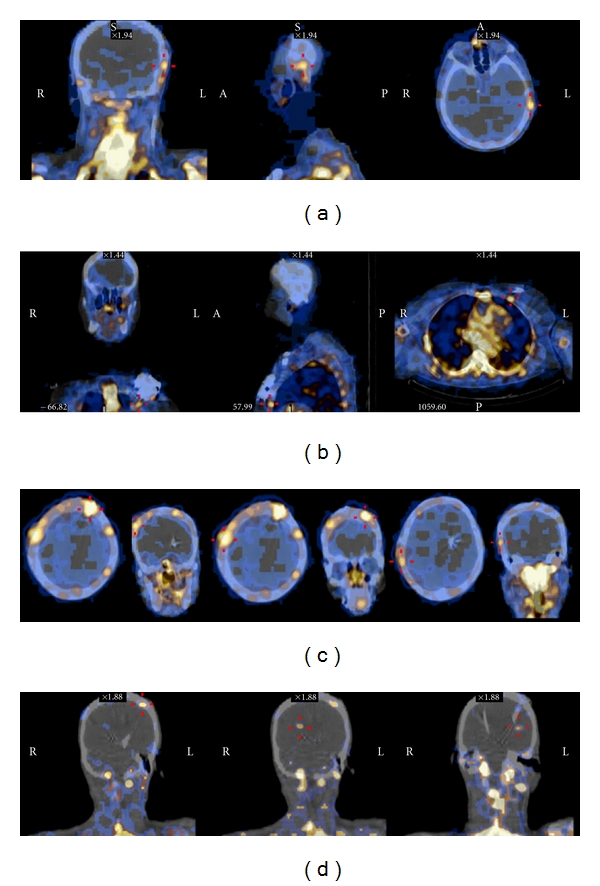
^
99m^Tc-sulesomab immunoscintigraphy (SPECT/CT fusion images). (a) Three plane images of Patient 2, showing focal tracer uptake restricted to the subcutaneous left temporal area, corresponding to the connector site. (b) Three plane images of Patient 7, showing left chest wall focal tracer uptake, in the area corresponding to the IPG pouch. (c) Axial and coronal images of Patient 1, showing diffuse tracer uptake along the extracranial trajectory of the right electrode. (d) Coronal images of Patient 8, showing left frontal and intracranial focal areas of tracer uptake, along the path of the electrodes.

In most patients, the results of the immunoscintigraphy matched the exact location of the wound dehiscence, with a focal pattern of uptake underlying the area of skin infection (Figures 1(a) and 1(b)). Nonetheless, a few exceptions are worth mentioning in greater detail. Firstly, both patients 1 and 5 showed an increased tracer uptake that was more diffuse than could be expected from clinical inspection of the wounds; specifically, these patients' scans showed a diffuse tracer uptake that included the frontal areas, albeit the absence of clinical signs of infection at these locations (Figure 1(c)). The presence of infection at these clinically unapparent locations was confirmed during surgery. As described in the following paragraph, these findings have had an impact on subsequent treatment strategies. Another exception was patient 4, whose scan did not show areas of increased uptake; of relevance, this was the only documented case with a negative microbiological examination of the wound serous exudate. Finally, patient 8 had unexpected intracranial uptake of the tracer (Figure 1(d)), which also influenced the course of treatment decisively. 

### 3.3. Therapeutic Approach according to ^99m^Tc-Sulesomab Immunoscintigraphy

#### 3.3.1. Frontal or Diffuse Tracer Uptake

The two patients who presented with a diffuse tracer uptake (patients 1 and 5) underwent a complete removal of the stimulation system, and with this approach the skin infections healed properly. It should be mentioned that both these patients had already undergone surgical removal of extracranial DBS components (IPG and extension cables) prior to the immunoscintigraphy, a procedure that had been ineffective. Therefore, in both patients with diffuse tracer uptake at multiple locations along the trajectory of extracranial hardware components, only complete removal of the stimulation system was effective in treating the hardware-related infection. 

Patient 6 had an abnormal focal tracer uptake in the left frontal area (corresponding to the burr hole site), and partial removal of the stimulation system including the left electrode was performed; the decision to remove the electrode was based on the fact that it was in direct contact with the pus, as could be observed during surgery. Although the right electrode was initially spared, it was later removed due to persistence of infection. Of note, the right electrode followed an extracranial trajectory to the left and was thus under the area of the original infection. 

Because patient 8 presented with intracranial tracer uptake, a complete removal of the stimulation system was undertaken, even though the patient had no clinical symptoms or signs of encephalitis, and cranial CT could not demonstrate areas of contrast enhancement suggestive of intracranial infection.

#### 3.3.2. Focal Retroauricular or Subclavicular Tracer Uptake

Patients 2 and 3 initially underwent a more conservative surgical approach, owing to the fact that wound dehiscence and tracer uptake were both localized to the retroauricular incision. Wound debridement was the treatment option for patient 2, based on the fact that no purulent exudate could be identified during surgery—performed after prolonged antibiotic therapy—despite the presence of tracer uptake at that location. Afterwards, due to recurrence of infection, IPG and extension cables were removed. Patient 3 had previously removed the IPG and extension cables and also underwent wound debridement, in addition to removal of the electrode protective caps, which contained a purulent exudate. However, due to persistence of infection, this patient later underwent bilateral electrode removal. 

The only patient with isolated subclavicular wound dehiscence (patient 7) had a focal tracer uptake on the chest wall and underwent removal of the IPG and extension cables, which proved to be a successful strategy.

#### 3.3.3. No Tracer Uptake

Finally, the only patient with a negative scan (patient 4) underwent wound debridement alone. Later, due to persistent skin erosion, in the absence of a purulent drainage or positive cultures, contralateral transposition of the extracranial DBS components was performed.

### 3.4. Follow-Up ^99m^Tc-Sulesomab Immunoscintigraphy and Reimplantation Surgery

Patients 2, 3, and 5 repeated the immunoscintigraphy scans in order to evaluate the feasibility of a reimplantation surgery. The scans were repeated on average three months after removal of DBS components. While patient 2 had a normal scan and underwent reimplantation of the IPG device, patients 3 and 5 maintained foci of increased ^99m^Tc-labeled sulesomab uptake, and reimplantation procedures were not attempted.

## 4. Discussion

Hardware infection in the context of DBS occurs at a rate of approximately 1–15%, depending on the series [5]. It usually represents a major management concern, often leading to the loss of the stimulation device. Several studies have analyzed the role of potential host risk factors in the development of skin complications following DBS implantation and failed to demonstrate a significant association [6, 10–13]. Nevertheless, because the rate of skin complications is higher in patients with PD than in patients with other diagnoses, it has been suggested that skin alterations in relation to PD itself could contribute to the occurrence of this adverse event [11, 12]. Also, a straight scalp incision and a period of externalization of the electrodes in dual-stage procedures have been suggested to increase the infection rate [14], although the latter has not been confirmed in other studies [10, 12, 13]. The utilization of Kinetra devices has also been implicated as a risk factor for the development of skin complications, due to its larger volume when compared to other types of IPG [11, 13]. Strategies to reduce the incidence of this complication have focused mainly on the development of smaller hardware components, reduction of operative times, and avoidance of temporary electrode externalization. It has also been suggested that local antibiotic application in addition to intravenous prophylaxis could lower the rate of infection after deep brain stimulator implantation [15]. While our knowledge on what factors associate with the development of skin complications does not progress to the point they can be prevented, it is important to focus on strategies that help clinicians decide the best treatment approach for each individual patient. Several authors consider that a conservative treatment trial with antibiotics may be warranted in certain cases [7, 14] while others find an early surgical approach more appropriate [13]. Nevertheless, the management of skin complications following DBS is still controversial, and no specific guidelines have been issued. 

In most published series, infection is finally treated with complete hardware removal, despite initial attempts at more localized treatment. The major issue about infection management is being able to identify which patients should undergo hardware removal and to what extent. Different treatment algorithms have been published trying to address this issue. The most consensual approach is that all hardware components that are in direct contact with the infection should be removed and that complete hardware removal should be used in patients with disseminated or multiple infectious foci [13, 16]. While generally agreeing with this strategy, there are still a large proportion of patients who fail the attempts to preserve the brain electrodes. Of relevance, our immunoscintigraphy results demonstrate that infection can be present in areas that look clinically intact (i.e., without skin erythema and swelling, erosion, or purulent discharge) due to the subcutaneous dissemination of infection from the primary site. In two of our patients, there was an unexpected tracer uptake in the frontal areas, despite the fact that the scalp wounds were clinically intact. These findings led to a more aggressive treatment approach than had initially been considered. Significantly, infection at these apparently intact locations was confirmed during surgery by the presence of pus. By allowing the accurate determination of the subcutaneous extent of the infection, which may not be clinically evident, ^99m^Tc-sulesomab scans could indeed influence the treatment strategy of post-DBS infection, with a diffuse or frontal uptake indicating the necessity of a complete removal of the stimulation system at presentation, thus avoiding conservative strategies that will most likely fail, in an unsuccessful attempt to partially preserve the hardware. The same strategy must apply to patients with intracranial tracer uptake (which was also an unexpected finding in our series), who should undergo immediate removal of DBS entirely. 

In those cases of circumscribed infection with a focal tracer uptake, hardware-related infection could potentially be treated with a more conservative approach. In particular, infections around the IPG or the extension cables could be successfully treated with electrode preservation [13]. The accurate determination of which hardware components are in direct contact with the infection can be accomplished during surgery, which explains the recommendation that the surgical treatment of hardware infection should depend on surgical findings [16]. But the fact that the ^99m^Tc-sulesomab immunoscintigraphy can help determine the actual extent of the infection in the preoperative stage can turn out to be very helpful in planning the surgical approach, as it can demonstrate all areas of involvement by the infectious process before the surgical procedure. 

The results of a follow-up ^99m^Tc-sulesomab immunoscintigraphy could also facilitate the decision of whether and when a reimplantation procedure would be appropriate. In other words, a normal follow-up scan can help identify which patients are more likely to be safely reimplanted at a later time. In contrast, patients who persistently demonstrate an abnormal uptake should not be considered for a reimplantation procedure, as the risk of recurrent infection is presumably high. 

To our knowledge, there is no data reporting ^99m^Tc-sulesomab immunoscintigraphy efficacy in the context of DBS. Our findings indicate that the scintigraphy correlates well with the presence of infection in this particular clinical setting, since all patients with a positive bacterial culture of the skin exudate had an abnormal scan, and the only patient with a negative culture had a normal scan. Also, the location of the foci of infection, as demonstrated by the presence of pus during the surgical procedures, matched the location of the tracer uptake, even in those cases with clinically unapparent infection. This indicates that ^99m^Tc-sulesomab immunoscintigraphy is a reliable method to detect infection in the context of DBS, thus contributing to the assessment of patients with hardware-related infection. A final statement on the availability of the ^99m^Tc-sulesomab immunoscintigraphy should be made—this imaging modality should be available at every hospital that has access to nuclear medicine imaging, specifically a SPECT-CT scanner. Although the costs of the scintigraphy are still considerable (approximately 850 US dollars), it is likely cost effective. By allowing a better distinction between patients who should have immediate removal of the whole system and those who could undergo a more conservative approach, it has the potential to reduce the number of hospital admissions, surgical interventions, and prolonged antibiotic treatments that most patients endure while attempting to preserve the stimulation device.

## 5. Conclusion

**Figure 2:**
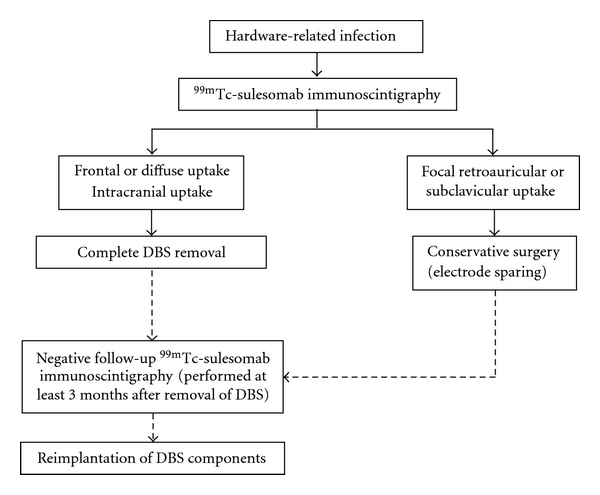
Proposed algorithm for the management of hardware-related infection.

Although only preliminary data is available in a small number of patients and more extensive followup is desirable, ^99m^Tc-sulesomab immunoscintigraphy seems to correlate well with the presence and extent of infection and therefore has a potential role in the management of skin complications following DBS surgery. It is particularly helpful in discriminating between patients who should remove DBS entirely at presentation and those in whom a conservative approach is more likely to succeed, thus complementing clinical and surgical assessments. Also, ^99m^Tc-sulesomab immunoscintigraphy has the potential to be very useful in the determination of which patients should be considered for reimplantation surgery. Finally, we propose an algorithm for the management of infection following DBS, based on the different patterns of tracer uptake in the ^99m^Tc-sulesomab immunoscintigraphy (Figure 2). 

## Figures and Tables

**Table 1:** Patients clinical description.

	Patient 1	Patient 2	Patient 3	Patient 4	Patient 5	Patient 6	Patient 7	Patient 8
Gender	F	M	M	F	F	M	F	M
Age at DBS	64	69	69	67	64	68	63	66
Site of skin erosion or infection	RetroauricularLeft frontal	Retroauricular	Retroauricular	Retroauricular	Retroauricular cervical	Left frontal	Subclavicular	Subclavicular initially, then left frontal
Culture	*S. aureus*	*S. aureus*	*S. aureus*	—	*S. aureus*	Unavailable	*S. mitis + S. oralis*	*S. aureus*
Number of previous surgeries in attempt to control infection	7 (including two exchanges of DBS components)	0	1	0	1	0	1	3
Result of previous surgical interventions	Removal of IPG + extension cables + left electrode	—	Removal of IPG + extensions cables	—	Removal of IPG + extensions cables	—	Hematoma evacuation + IPG repositioning	Removal of IPG + extensions cables

**Table 2:** ^
99m^Tc-sulesomab immunoscintigraphy results and patient outcome.

	Patient 1	Patient 2	Patient 3	Patient 4	Patient 5	Patient 6	Patient 7	Patient 8
^ 99m^Tc-sulesomab immunoscintigraphy	Diffuse uptake (right frontal, left parietal and retroauricular)	Focal uptake (retroauricular)	Focal uptake (retroauricular)	No uptake	Diffuse uptake (bilateral frontal andretroauricular)	Focal uptake (left frontal)	Focal uptake (subclavicular)	Focal uptake (left frontal + intracranial along the electrodes trajectory)
Findings during surgery	Purulent exudate over the right burr hole cap and along the extracranial trajectory of right electrode	Serous exudate under retroauricular wound	Purulent exudate around electrode protective caps	Serous exudate under retroauricular wound	Purulent exudate over the burr hole caps and along the extracranial trajectory of electrodes	Purulent exudate over left burr hole cap and around the left electrode	Purulent exudate on the IPG pouch	Purulent exudate over burr hole caps and around electrodes
Surgical procedure	Removal of right electrode	Wound debridement	Wound debridement Removal of electrode protective caps	Correction of wound dehiscence	Bilateral electrode removal	Removal of left electrode + IPG + extension cables	Removal of IPG + extension cables + left electrode (damaged during surgery)	Bilateral electrode removal
Patient outcome	Healed infectionUnilateral salvage pallidotomy	No healed infection and subsequent removal of IPG + extension cablesHealed infectionNormal scanRe-implantation surgery (8 months follow-up)	No healed infection and subsequent removal of electrodesHealed infectionNo reimplantation surgery (positive scan + dementia)	Persistent skin erosion at connector site and subsequent contralateral transposition of IPG and connector (3 months follow-up)	Healed infectionNo reimplantation surgery (positive scan)	No healed infection and subsequent removal of right electrodeHealed infectionNo reimplantation surgery yet	Healed infectionNo reimplantation surgery (patient refused)	Healed infectionNo reimplantation surgery (dementia)
